# MiR-937-3p promotes metastasis and angiogenesis and is activated by MYC in lung adenocarcinoma

**DOI:** 10.1186/s12935-022-02453-w

**Published:** 2022-01-15

**Authors:** Zijian Ma, Ganyi Chen, Yiqian Chen, Zizhang Guo, Hao Chai, Yu Tang, Lin Zheng, Ke Wei, Chunfeng Pan, Zhifei Ma, Yang Xia, Aiping Zhang

**Affiliations:** 1 Department of Thoracic Surgery, Nanjing First Hospital, Nanjing Medical University, Nanjing, China; 2 Department of Thoracic Surgery, The First Affiliated Hospital of Nanjing Medical University, Nanjing, China; 3 Department of Thoracic and Cardiovascular Surgery, Nanjing First Hospital, Nanjing Medical University, 68 Changle Road, Nanjing, 210029 China; 4 Department of Thoracic and Cardiovascular Surgery, The First Affiliated Hospital of Nanjing Medical University, 300 Guangzhou Road, Nanjing, 210029 China

**Keywords:** Lung adenocarcinoma, MYC, miR-937-3p, SOX11, EMT

## Abstract

**Background:**

Non-small cell lung cancer (NSCLC) is still one of the diseases with the highest mortality and morbidity, and lung adenocarcinoma (LUAD) accounts for more than half of all NSCLC cases in most countries. miRNA can be used as a potential biological marker and treatment for lung adenocarcinoma. However, the effect of miR-937-3p to the invasion and metastasis of LUAD cells is not clear.

**Methods:**

miRNA microarray is used to analyze the expression of miRNA in lung adenocarcinoma tissue. Transwell migration, Wound-healing assay and Western blot analysis are used to analyze cell migration, invasion and epithelial-mesenchymal transition (EMT) capabilities. Tube formation is used to assess angiogenesis ability. In addition, dual luciferase reporter gene detection is used to identify the potential binding between miRNA and target mRNA. In vivo experiments were performed on male NOD/SCID nude mice by tail vein injection to establish a transplanted tumor model. The CHIP experiment is used to verify the transcription factors of miRNA.

**Result:**

In our study, miR-937-3p was high-regulated in LUAD cell lines and tissues, and its expression level was related to tumor progression. We found that miR-937-3p high-expression has an effect on cell invasion and metastasis. In molecular mechanism, miR-937-3p causes SOX11 reduction by directly binding to the 3′-UTR of SOX11.In addition, MYC affects miR-937-3p transcription by binding to its promoter region.

**Conclusions:**

Our research shows that miR-937-3p is mediated by MYC and can control the angiogenesis, invasion and metastasis of LUAD by regulating SOX11, thereby promoting the progress of LUAD. We speculate that miR-937-3p can be used as a therapeutic target and potential biomarker for LUAD.

**Supplementary Information:**

The online version contains supplementary material available at 10.1186/s12935-022-02453-w.

## Introduction

Lung adenocarcinoma (LUAD) is the main source of non-small cell lung cancer (NSCLC), and NSCLC is still the leading cause of death from malignant tumors. Every year, there are 2.09 million new lung cancer patients and 1.76 million deaths [1]. Lung cancer is mainly NSCLC (85%), and NSCLC is further divided into squamous cell carcinoma, adenocarcinoma and large cell carcinoma according to the classification of WHO [2]. Despite the recently continuous improvement of traditional treatment methods such as surgical therapy, radiotherapy, chemotherapy, and targeted therapy, the survival probability is still low, about 17% [3, 4]. In the development of tumors, the abnormal expression of oncogenes and tumor suppressor genes, such as changes in various regulatory factors, are currently important anti-tumor targets and have become hotspots for research.

miRNA is a small RNA composed of approximately 22 nucleotides, which binds to partial complementary sequences in the 3′-untranslated region (3′-UTR) of mRNA and negatively affects post-transcriptional regulation [5, 6]. miRNAs that are abnormally expressed in tumors may play a role in promoting or suppressing tumors. miR-760 can inhibit the expression of ROS1, thereby inhibiting multiple cell pathways such as STAT3, PI3K/AKT/mTor to regulate proliferation and inhibit in lung cancer cell lines tumor growth [7]. miR-1260b inhibits SOCS6 and activates the KIT pathway to inhibit the proliferation and the apoptosis of NSCLC [8]. In recent years, several emerging studies have reported that miRNAs are involved in cell proliferation, differentiation and apoptosis and other tumor progression processes [9, 10].

miR-937 plays different roles in the development of many diseases. Studies have found that peripheral blood miR-937 can target AMPKα as a biomarker of metabolic disorders. miR-937 is highly expressed in colon cancer, breast cancer and hepatocellular carcinoma, and promotes tumor development to predict the poor prognosis of patients. We found that miR-937-3p is usually highly expressed in LUAD patients, especially in LUAD with metastasis. In in vitro experiments, miR-937-3p promotes cell invasion and metastasis. This study also showed that SOX11 is the downstream gene target of miR-937-3p through bioinformatics prediction, western blot results and dual luciferase reporter gene analysis. SOX11 is a tumor suppressor gene. Up-regulation of SOX11 hinders the invasion and proliferation of LUAD cells. MicroRNA-223-3p can promote the proliferation and invasion of ovarian cancer cells by targeting SOX11 expression [11].

We speculate that miR-937-3p plays a role in promoting cancer in lung adenocarcinoma and promotes the development and metastasis of cancer. In addition, we found through western blot that miR-937-3p can target SOX11 to activate the PI3K/AKT pathway, thereby promoting NSCLC angiogenesis and mediating NSCLC metastasis. We have reported a new pathway in LUAD, where MYC-regulate miR-937-3p targets SOX11 and enhances the angiogenesis, invasion and metastasis of LUAD.

## Methods

### Clinical samples and cancer cell lines

A total of 60 NSCLC patients with cancerous tissue and adjacent tissues were tested in this study. The sampled patients all haven’t received radiotherapy or chemotherapy before surgery, and all of them did not have cancer before. All patients underwent surgery in the First Affiliated Hospital with Nanjing Medical University from October 2015 to July 2020. Their specimens were kept in liquid nitrogen after they were isolated and before use. Written consent was obtained from all participants. This study has been approved by the ethics committee of our hospital. A total of 6 human LUAD cell lines (A549, H1299, H358, PC9 and H1395) and one human bronchial epithelial-like cell (16HBE) were provided by the Shanghai Academy of Sciences. All the above cells were cultured in 10% fetal bovine serum (Gibco, NY, USA) + RPMI-1640 medium (Gibco, NY, USA), which was supplemented with 1% penicillin/streptomycin (Gibco, NY, USA), in a humidified cell incubator containing 5% carbon dioxide at 37 °C.

### ISH staining

In this study, in situ hybridization (ISH) was used to check the expression level of miR-937-3p in human LUAD tissue and adjacent tissues. Paraffin-embedded tissue was hydrated and dewaxed using Proteinase K digestion (20 µg / ml) for 20 min at 36 °C. Tissue was washed three times with PBS and incubated with hsa-miR-937-3p probe pre-mixed solution (10 ng/µl) (Servicebio, Wuhan, China) at 37 °C overnight. Anti-DIGHRP was added for 30 min with 3,3-Diaminobenzidine (DAB) solution was stained, and nuclei were stained with hematoxylin. Photos are taken under a microscope. The probe sequence for miR-937 ISH is in Additional file 4: Table S1.

### Quantitative reverse transcription PCR

In order to determine the expression level of miR-937-3p in LUAD tissues and cells, total RNA was isolated from tissues and cells using RNA-Quick Purification Kit (YiShan bio, Shanghai, China) according to the instructions. CDNA was generated using total RNA and PrimeScriptRT reagent (Takara, Kusatsu, Japan), and qRT-PCR was performed on an ABI StepOne Plus system (Applied Biosystems, CA) using SYBR Green Master Mix II (Takara). U6 and GAPDH were used as the internal controls to normalize SOX11 and miR-937-3p. We used the 2 − ΔΔCT method to quantify the relative levels of miR-937-3p and SOX11 mRNA. Every sample was performed in triplicate.

### Cell transfection

Lentivirus (Lv-hsa- miR-937-3p-mimics, Lv-has-miR-937-3p inhibitor, Lv-NC) was purchased from Keruisi (Jiangsu, China) and transfected according to the manufacturer’s instructions. Plasmids SOX11, empty vector, siRNA-SOX11 and siRNA-MYC were purchased from Gene Pharma (Shanghai, China). Transfection was performed using the lipofectamine 3000 reagent (Invitrogen,CA,USA) according to the manufacturer’s instructions. The sequence for transfection is in Additional file 4: Table S1.

### Wound-healing assay

Approximately 4 × 10^5^ cells were evenly spread into a 6-well plate. The 6-well plate was washed with 2 ml PBS and then scratched in the Central axis by a sterile 200 µl sterile plastic tip. It was washed three times with PBS and re-cultured with 2 ml of 2% FBS-containing medium. 24 h later, the distance between the scratches was measured with an inverted microscope. Each sample was performed in triplicate.

### Transwell assay and recruitment assay

Approximately 3 × 10^4^ cells in serum-free medium (400 µl) were seeded into the upper Transwell chamber for migration assay. The invasion assays were performed using 3 × 10^5^cells in serum-free media transferred on the upper chamber of an insert coated with Matrigel (BD Biosciences). Transwell migration and invasion assays have used 8.0 μm Transwell Permeable Supports (Corning, New York, USA). Add 800 µl of RPMI-1640 medium containing 10% FBS to the lower chamber and incubate at 37 °C for 1 day. After 24 h, use a moistened cotton swab to remove the remaining cells on the upper surface. At room temperature, we use paraformaldehyde and crystal violet (Beyotime) to fix and stain the cells on the lower surface of the membrane for 30 min. The number of stained cells in five randomly selected fields was counted with an optical microscope (× 100). Each sample was performed in triplicate.

### Tube formation

Approximately 1 × 10^5^ HUVEC cells were resuspended with cellular supernatant in each well of a 24-well plate containing 250 µl Matrigel (BD Bioscience, USA). Tube formation was incubated at 37 °C for 6 h. Use a microscope (×100) to calculate the tube length for each field of view. Each sample was performed in triplicate.

### Luciferase reporter assay

Insert the 3′-UTR sequence or mutation sequence of SOX11 and the predicted target site into the pmir-GLO-promoter vector (Goruse biotechology, Nanjing, China). Cells were transfected with the WT or Mut-plasmids by Lipofectamine 3000 reagents (Invitrogen, USA). Using luciferase Assay System (Promega, USA) to evaluate the activity after transfection.

### Western blotting

RIPA reagent (Beyotime, Shanghai, China) containing 10 µg/ml PMSF (Beyotime, Shanghai, China) and 10 µg/ml Phosphatase inhibitor was added to LUAD specimens or cells and then placed on ice for 15 min to extract the proteins. The protein lysates were separated by SDS-PAGE and then transferred to polyvinylidene difluoride (PVDF) membrane. Primary antibodies used in Western blotting were GAPDH (Cell Signaling Technology, 5174), EMT Antibody Sampler Kit (Cell Signaling Technology, # 9782), SOX11(Abcam, ab229185), MYC (Cell Signaling Technology #9402).

### Animal assay

Eight male NOD/SCID mice (4~6 weeks old) were purchased from the Animal Center of Nanjing Medical University, which is approved by the Animal Ethics Committee of Nanjing Medical University. Eight male NOD / SCID mice (4~8 weeks old) were randomly divided into 2 groups (4 in each group). 80 µl of cell suspension containing 3 × 10^7^ lv-miR-937-3p cells or lv-miR-control-cells was injected into each mouse through the tail vein, which was used for lung colonization. Eight weeks later, the mice were euthanized by cervical dislocation, and the transfer node was checked using the IVIS Lumina II system.

### IHC

The collected metastatic tumors were fixed with 4% paraformaldehyde and embedded in paraffin. All paraffin sections were deparaffinized and rehydrated the next day. Tumor tissue sections and incubations were incubated with antibodies SOX11 (Abcam, ab229185) and MMP2 (Abcam ab86607) at 4 °C overnight. Wash the sections with PBS the next day, and then incubate them with HRP goat anti-rabbit IgG (Abcam, ab205718) for 1 h at room temperature, and stained with DAB solution. Hematoxylin is used for nuclear staining.

### IF

The cells were spread on a six-well plate cell climbing slide. The cell fusion degree was about 70%, then the cells were fixed with paraformaldehyde, penetrated with 0.2% Triton X-100 for 20 min, and blocked with 1% bovine serum albumin (BSA) for 60 min. EMT Antibody SamplerKit (Cell Signaling Technology, 9782) was diluted 1:400 and incubated at 4 ℃ overnight. Anti-rabbit IgG (H + L) (Cell Signaling Technology, 8889S) was diluted to 1: 250 in 1% BSA PBS and incubated for 1 h. Pictures were taken under a fluorescence microscope.

### CHIP assay

Performed using the ChIP assay kit (Millipore, USA) according to the manufacturer’s instructions. About 5 × 10^7^ cells were collected and cross-linked with 1% formaldehyde (Bio-Rad, CA, USA) for 9 min. By sonicating the lysate, the chromatin is cut into fragments of 100–500 bp in size. After shedding, the insoluble matter was separated by centrifugation, and 100 µl of DNA / protein complex was used as the input. MYC (Cell Signaling Technology #9402), normal rabbit IgG antibody (Cell Signaling Technology, 2729), and A/G protein beads were incubated overnight at 4 ℃. After 4 h’s incubation at 65 °C, the input and the sample were cross-linked in reverse. Then, DNA was extracted from the sample with phenol / chloroform (Invitrogen), and promoter binding was assessed by PCR.

### Statistical analysis

All statistical were performed by GraphPad Prism software 8.0 and SPSS 22.0. The *P*-values were analyzed under Student’s *t* test and Spearman’s test. If p < 0.05, it is considered statistically significant. (*P < 0.05; **P < 0.01; ***P < 0.001).

## Results

### miR-937-3p is associated with LUAD metastasis

**Fig. 1:**
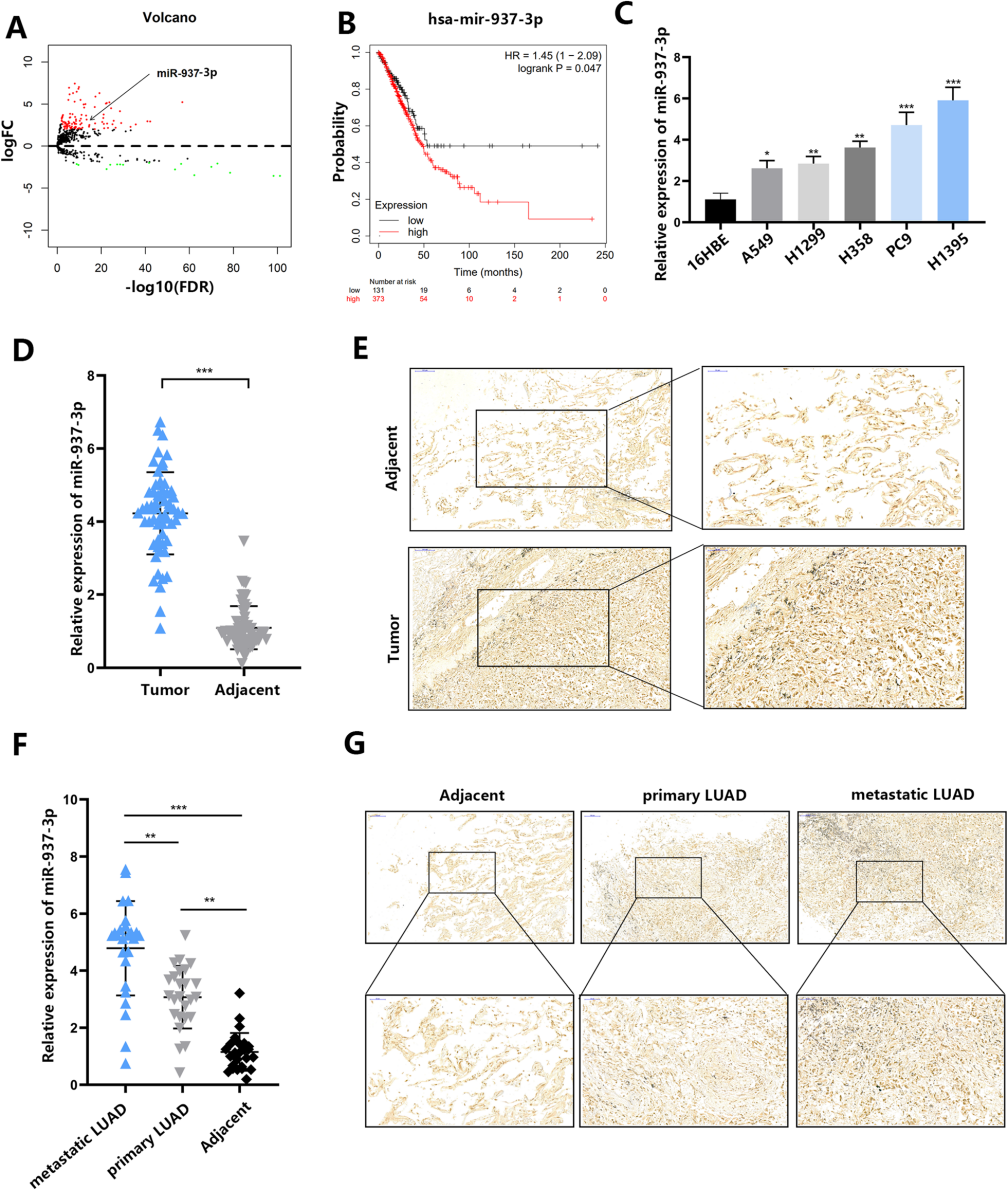
miR-937-3p is associated with LUAD metastasis. **A** The volcano plot shows the difference between miRNAs in the tissues of healthy individuals and LUAD patients in the TCGA database. The miRNAs were classified based on fold change (log2fc) between the two groups. **B** The relationship between miR-937-3p and the survival of lung cancer patients (n = 131 in the miR-937-3p-low group and n = 373 in the miR-937-3p-high group). **C** The miR-937-3p expression level in NSCLC cell lines and paired 16HBE was investigated by qRT-PCR. **D** The miR-937-3p expression level in 60 NSCLC tissues and paired adjacent tissues was investigated by qRT-PCR. **E** ISH for miR-937-3p in tumor tissue and corresponding paracancerous tissues. **F** qRT-PCR analysis of miR-937-3p expression in primary LUAD tissues with or without metastasis and adjacent tissues (25 adjacent,25 without metastasis and 25 with metastasis). **G** ISH for miR-937-3p in primary LUAD tissues with or without metastasis and adjacent tissues. The data are represented as mean ± SD. (*P < 0.05; **P < 0.01; ***P < 0.001)

We obtained the data of miRNA microarray from TCGA. We use edgeR to detect significantly differentially expressed miRNAs (DEmiRNA). First, we explored the differential expression of miRNA in 792 LUAD tumors and 203 normal tissues. Using standard filter analysis (absolute fold change > 1.5 and FDR value <0.01), 128 miRNAs between normal and LUAD tissues were explored (Fig. 1A, Additional file 1: Fig. S1A). Through online survival prognosis analysis (http://kmplot.com/analysis/), we found that four highly expressed miRNAs (miR-937-3p, miR-195, miR-142, miR-345) have important prognosis for survival (Fig. 1B, Additional file 2: Fig. S2A). Compared with primary LUAD, miR-937-3p was most prominent in the metastatic LUAD (10 with metastasis, 10 with non-metastatic) (Additional file 2: Fig. S2B). So we selected miR-937-3p for further study. Next, we found that miR-937-3p expression level was higher in tumor cell lines than in 16HBE cells (Fig. 1C). Among them, the expression of miR-937-3p was highest in H1395 cells, while the expression of miR-937-3p in A549 cells was the lowest. Besides, we examined the expression of miR-937-3p in LUAD tissues and adjacent tissues (n = 60) (Fig. 1D). The expression level of miR-937-3p was significantly up-regulated in LUAD tissues, indicating that miR-937-3p may be an oncogene involved in the development of LUAD. Beside, ISH showed that miR-937-3p was mainly expressed in malignant epithelial cells compared to adjacent tissues (Fig. 1E). Interestingly, we found that the expression of miR-937-3p in LUAD with metastasis was higher than that in LUAD without metastasis, which was further verified by ISH (Fig. 1F, G). These results all indicate that miR-937-3p may be involved in the occurrence and metastasis of LUAD.

### miR-937-3p affects cell angiogenesis, invasion and metastasis

**Fig. 2:**
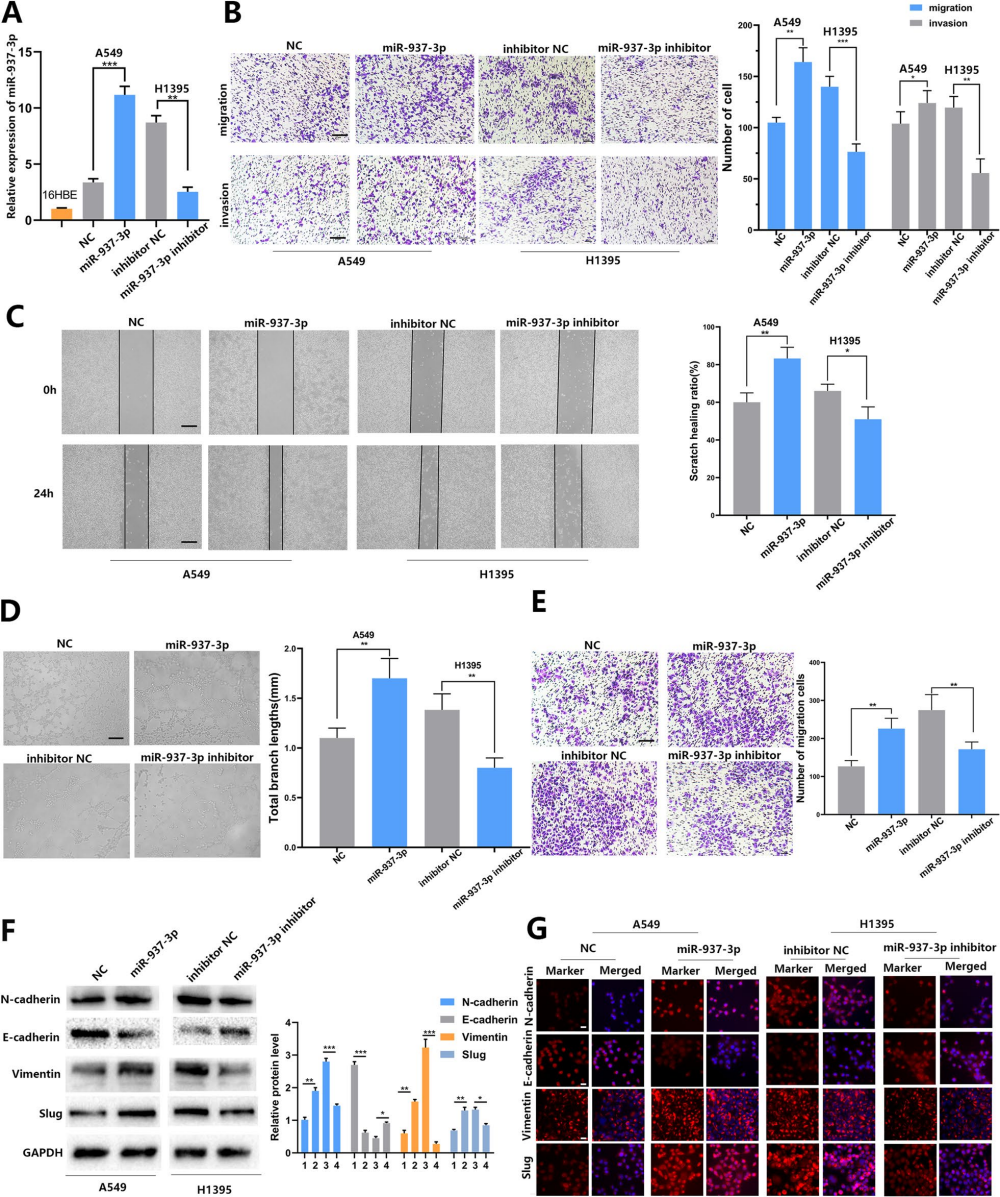
The effects of miR-937-3p on LUAD cell metastasis and angiogenesis. **A** After transfection and the expression level of miR-937-3p was detected by qRT-PCR. **B** Transwell assay was employed to determine invasion and migration ability of transfected LUAD cells; scale bars, 50 μm. **C** Wound healing assay was used to detect the cell migration ability; scale bars, 100 μm. **D** Effect of miR-937-3p expression on angiogenesis; scale bars, 200 μm. **E** The role of miR-937-3p in the recruitment of HUVECs; scale bars, 50 μm. **F**, **G** Effect of miR-937-3p on EMT was tested by western blot and immunofluorescence analyses. 1: NC;2: miR-937-3p;3: inhibitor NC;4: miR-937-3p inhibitor; The nucleus stained with DAPI was blue; scale bars, 10 μm. The data are represented as mean ± SD. (*P < 0.05; **P < 0.01; ***P < 0.001)

We selected H1395 cells as high-expressing cells and A549 cells as suppressor cells. Compared with normal A549 cells, the expression of miR-937-3p was up-regulated in cells transfected with lv-miR-937-3p, while the expression of miR-937-3p in H1395 cells transfected with lv-miR-937-3p-inhibitor was down-regulated (Fig. 2A). The effects of miR-937-3p on cell invasion, metastasis and angiogenesis were measured by Transwell, scratch experiments and tube formation. It was found that high expression of miR-937-3p promotes the invasion, metastasis and angiogenesis of A549 cells, while low expression of miR-937-3p in H1395 cells exerted an inhibition effect (Fig. 2B–D). At the same time, we use PCR to measure the level of miR-937 in HUVECs after treatment (Additional file 2: Fig. S2C). In order to further explore the effect of miR-937-3p on angiogenesis, we have conducted a recruitment experiment and found that high-expression of miR-937-3p promotes HUVEC migration, while low expression has the opposite effect (Fig. 2E). We guessed whether miR-937-3p would generate EMT and cause the invasion and metastasis of LUAD. For this reason, we conducted Western Blotting and Immunofluorescence experiments for further verification. Through Western blot and IF, we found that the expression of epithelial cell markers (E-cadherin) in miR-937-3p overexpression cell lines was down-regulated compared with NC cell lines, and the expression of mesenchymal cell markers (vimentin, Slug and N-catenin) are upregulated. Whereas low expression of miR-937-3p in H1395 cells exerted an inhibition effect (Fig. 2F, G).

### SOX11 is the direct target gene of miR-937-3p in LUAD cell lines

**Fig. 3:**
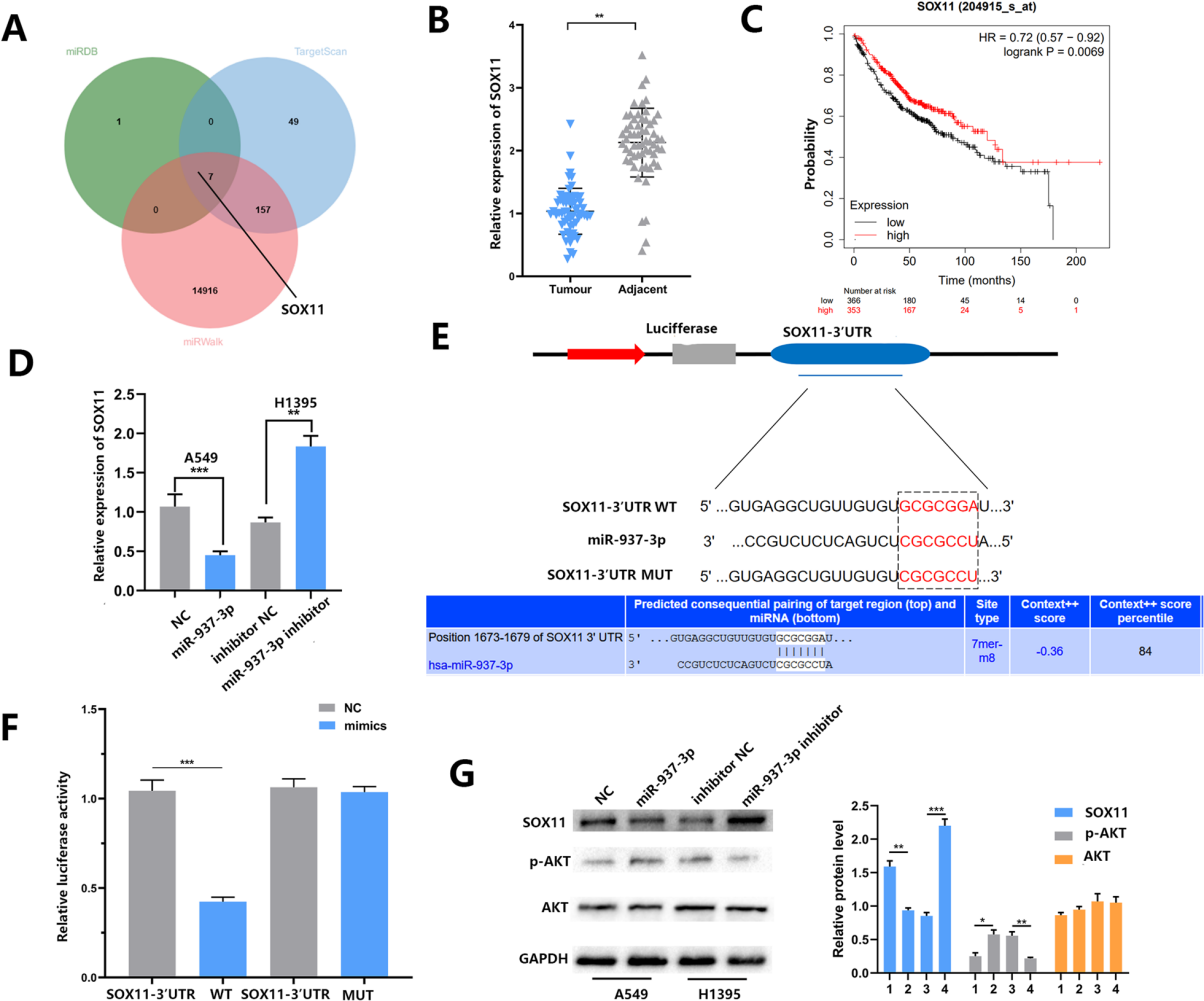
SOX11 is a direct downstream miR-937-3p target in LUAD cells. **A** Predicating candidate gene targets by intersecting from three different bioinformatics websites (TargetScan, miRDB and miRWalk). **B** The SOX11 expression level in 60 LUAD tissues and paired adjacent tissues was investigated by qRT-PCR. **C** The relationship between SOX11 and the survival of LUAD patients (n = 366 in the miR-937-3p-low group and n = 353 in the miR-937-3p-high group). **D** A negative regulatory effect of miR-937-3p on SOX11 was tested by qRT-PCR. **E** Predicted miR-937-3p seed region at the 3′- UTR of SOX11. **F** Luciferase activity was analyzed in LUAD cells co-transfected with miR-937-3p-mimics or negative control with pmirGLO-SOX11-Wt or pmirGLO-SOX11-Mut. **G** The expression levels of SOX11 protein and AKT pathway related proteins in the transfected A549 and H1395 cells were analyzed by Western blot. 1: NC;2: miR-937-3p;3: inhibitor NC;4: miR-937-3p inhibitor; GAPDH was used as a control. The data expressed as the mean ± SD. (*P < 0.05; **P < 0.01; ***P < 0.001)

In order to explore the downstream regulatory mechanism of miR-937-3p promoting LUAD metastasis and angiogenesis, we intersected the outputs of three online bioinformatics databases (TargetScan, miRDB, and miRWalk) to predict candidate gene targets. Based on these results, we found that SOX11 was the target gene of miR-937-3p (Fig. 3A). We found that SOX11 is low in cancer tissues of LUAD patients and is related to survival prognosis. Through online survival analysis, we found that in lung adenocarcinoma, patients with low SOX11 expression have a better prognosis (Fig. 3B, C). At the same time, in order to evaluate the response of SOX11 expression to miR-937-3p, PCR results showed that miR-937-3p has a negative regulatory effect on SOX11. Up-regulating miR-937-3p has resulted in decreased SOX11 expression, while down-regulating miR-937-3p has resulted in increased SOX11 expression (Fig. 3D). Therefore, we have performed a luciferase reporter assay to explore whether the 3′-UTR of SOX11 is a direct target of miR-937-3p. The use of vectors containing site mutation sequences is not affected by miR-937-3p, but the relative luciferase intensity of cells with miR-937-3p and SOX11 3′-UTR plasmids is significantly reduced (Fig. 3E, F). Thus, SOX11 was confirmed as a direct downstream target of miR-937-3p. In addition, we speculate that SOX11 can mediate the activation of certain signaling pathways and promote the metastasis and tube formation of NSCLC. We examined several classic signaling pathways, and the results showed that miR-937-3p can up-regulate the phosphorylation level of AKT (p-AKT). In summary, miR-937-3p can regulate SOX11 expression and downstream PI3K/AKT signaling (Fig. 3G).

### SOX11 overexpression reversed the effect of miR-937-3p on the malignant progress of LUAD cells

**Fig. 4:**
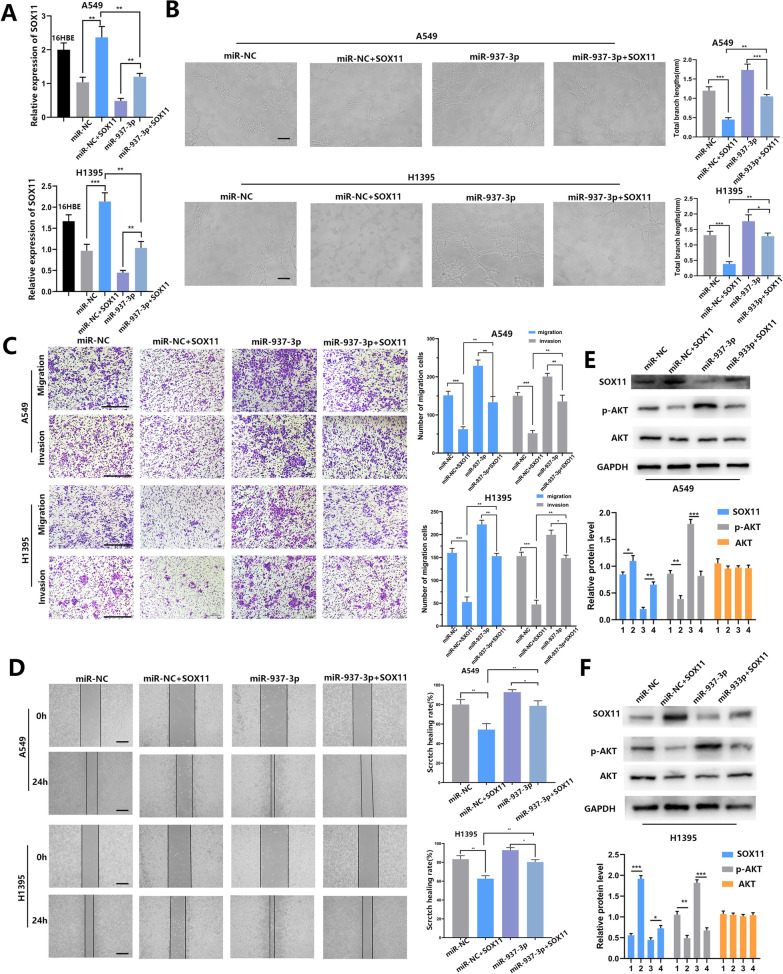
SOX11 overexpression reversed the effect of miR-937-3p on the malignant progress of LUAD cells. **A** After co-transfection and the expression level of SOX11 was detected by qRT-PCR. **B** The roles of promoting angiogenesis of miR-937-3p was attenuated by high expression of SOX11; scale bars, 100 μm. **C**, **D** Transwell assays and wound-healing assay detected that SOX11 attenuated the inhibitory effects of miR-937-3p; scale bars, 200 μm. **E**, **F** The expression levels of SOX11 protein and AKT pathway related proteins after SOX11 plasmid transfection were analyzed by Western blotting in co-transfected cell lines. 1: miR-NC;2: miR-NC+SOX11;3: miR-937-3p;4: miR-937-3p+SOX11; The data expressed as the mean ± SD. (*P < 0.05;**P< 0.01; ***P < 0.001)

In order to further confirm the role of SOX11 in miR-937-3p-affected angiogenesis, invasion and metastasis, we performed a rescue test. We stably transfected A549/H1395 cells with the SOX11 plasmid, and detected the expression level of SOX11 in transfected cells by qRT-PCR (Fig. 4A). Subsequently, tube formation assays, transwell experiments and scratch experiments were performed on co-transfected cells. The effect of up-regulation of miR-937-3p on the angiogenesis of LUAD cells was reversed by the high expression of SOX11 (Fig. 4B). Similarly, it was also found that up-regulation of SOX11 inhibited invasion and metastasis of cells (Fig. 4C, D). At the same time, we found through western blot that SOX11 overexpression can rescue the decline in SOX11 expression induced by lv-miR-937-3p. In addition, the rescue effect also changed the level of p-AKT (Fig. 4E, F). In summary, SOX11 overexpression reversed the effect of miR-937-3p on the malignant progress of LUAD cells. In order to further verify our conjecture, it was found by Western blot that the overexpression of SOX11 can rescue the decrease in SOX11 expression induced by lv-miR-937-3p. In addition, the rescue effect also changed the level of p-AKT (Fig. 4E, F). In conclusion, we found that SOX11 overexpression reversed the effect of miR-937-3p on the malignant progression of LUAD cells.

### miR-937-3p regulates LUAD cell metastasis and angiogenesis in vivo

**Fig. 5:**
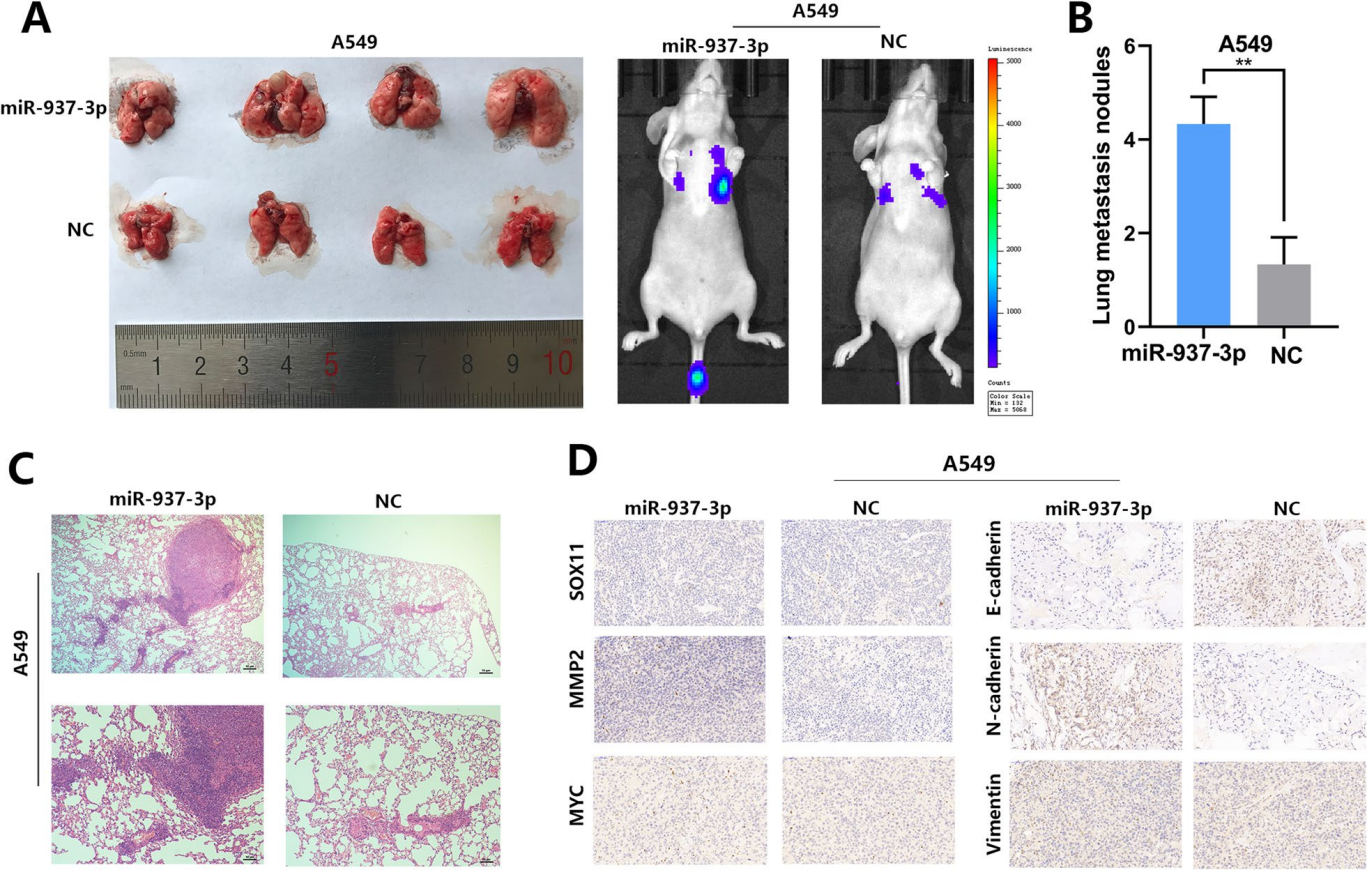
miR-937-3p promotes metastasis via targeting SOX11. **A**, **C** Visualization of the entire lung and HE-stained lung sections after injecting transfected NSCLC cells into nude mice. **B** The number of lung metastatic sites was counted under the microscope. **D** SOX11 and MMP2 expression levels the samples collected from tumor nodules were analyzed by IHC; scale bars, 50 μm. The data expressed as the mean ± SD. (*P < 0.05; **P < 0.01; ***P < 0.001)

We injected A549 cells transfected with Lv-miR-937-3p into the tail veins of 8 male mice to further verify the effect of miR-937-3p on the metastasis and angiogenesis of LUAD cells in vivo. After 8 weeks, the mice were sacrificed and their lung tissues were taken out and the metastatic nodules on the lung surface were counted. Compared with the control group, Lv-miR-937-3p increased the number and volume of metastatic nodules (Fig. 5A, B). The results showed that the increase of miR-937-3p significantly increased the number and size of lung metastases by H&E staining (Fig. 5C). We further conduct IHC experiments on the metastatic tumors. MMP2 is known to be involved in the related processes of blood vessel formation and invasion and metastasis. In addition, the expression of SOX11 in Lv-miR-937-3p group was reduced, and the positive rate of MMP2 in Lv-miR-937-3p group was higher than that in control group (Fig. 5D). Therefore, our in vivo data complements the results of miR-937-3p in vitro function studies.

### MYC was an upstream regulator of miR-937-3p by directly binding to its promoter region

**Fig. 6:**
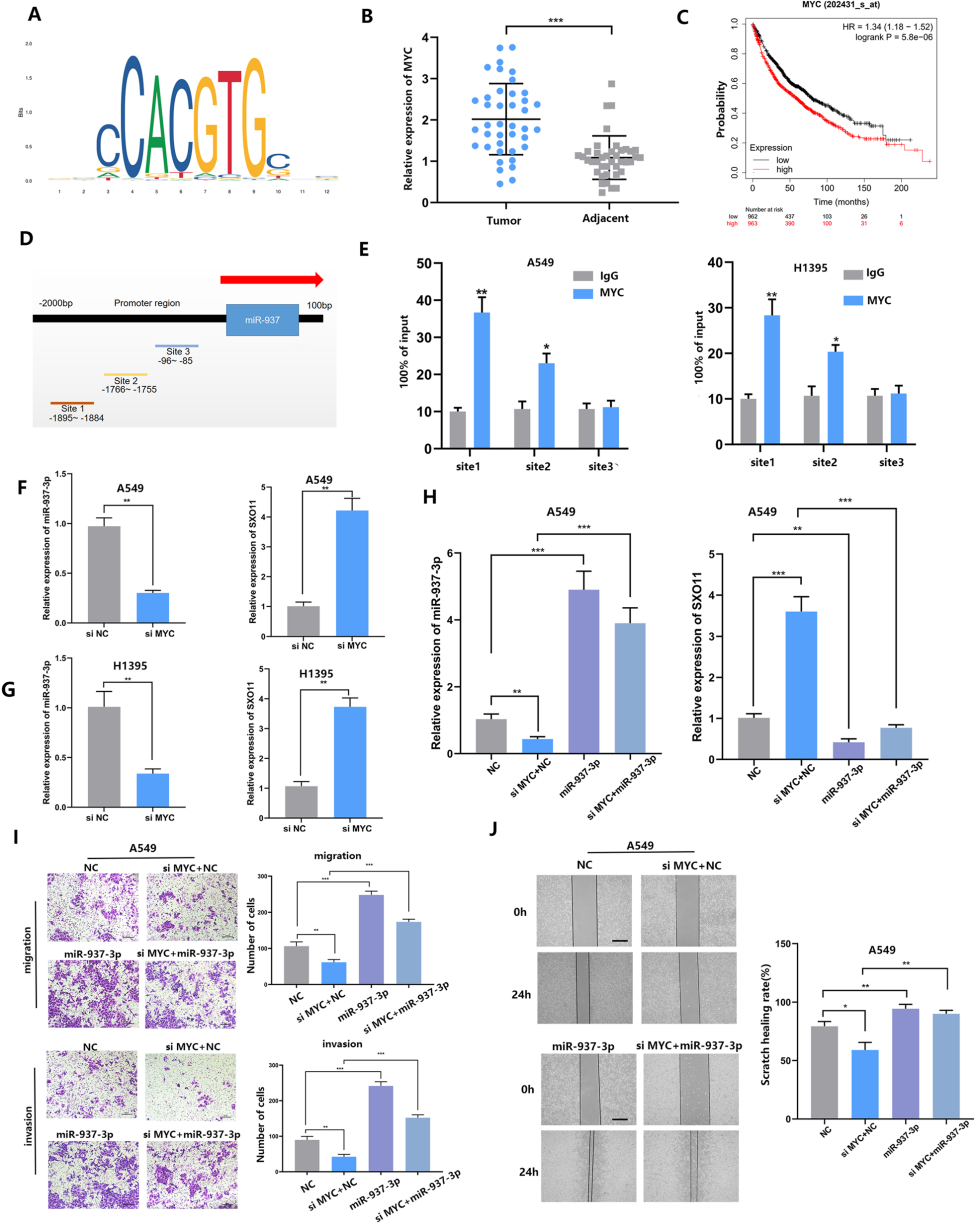
MYC was an upstream regulator of miR-937-3p. **A** Binding motif of MYC in the promoter of miR-937-3p, predicted by JASPAR (http://jaspar.genereg.net/). **B** The MYC expression level in 40 LUAD tissues and paired adjacent tissues was investigated by qRT-PCR. **C** MYC was closely related with survival probability. **D** miR-937-3p gene promoter region. **E** Chromatin immunoprecipitation (ChIP) assay indicated the MYC binds to the putative binding site upstream of miR-937-3p. **F**, **G **The relative expression levels of miR-937-3p and SOX11 responding downregulated MYC were detected by qRT-PCR. **H** Levels of miR-937-3p and SOX11 was accessed by qRT-PCR after co-transfection. **I**, **J **Migration and invasion was determined after co-transfected; scale bars, 50 μm; scale bars, 200 μm. The data expressed as the mean ± SD. (*P < 0.05; **P < 0.01; ***P < 0.001)

Since miR-937-3p functions as an onco-miRNA in LUAD, we hypothesized that miR-937-3p is likely to be activated by some pro-tumor factors. We selected 2000 sites upstream of miR-937-3p as regions where transcription factors may exist. Based on the JASPAR and UCSC databases, we found that multiple genes within the miR-937-3p promoter region may affect its transcription. Among them, MYC combined with miR-937-3p had the highest putative score (Fig. 6A, Additional file 2: Fig. S2E), and MYC expression was up-regulated in a variety of tumors, promoting tumorigenesis and development. We found that compared with adjacent tissues, MYC expression in LUAD is increased, and MYC is also related to survival prognosis (Fig. 6B, C). Therefore, we continue to verify the potential regulatory relationship between miR-937-3p and MYC, and choose the three highest scoring sites for CHIP experiments (Fig. 6D). ChIP experiments showed that MYC binds upstream of the putative binding site of miR-937-3p (Fig. 6E). At the same time, we used si-MYC expression to down-regulate the relative expression level of MYC (Additional file 2: Fig. S2F). After 36 h, the reduction of MYC expression can significantly reduce the level of miR-937-3p and increase the level of SOX11 (Fig. 6F, G). These results suggest that miR-937-3p is positively correlated with MYC in LUAD cells. First, after co-transfecting Lv-miR-937-3p or Lv-vector with si-MYC, we used PCR to detect the expression levels of miR-937-3p and SOX11 in LUAD cells (Fig. 6H, Additional file 3: Fig. S3A). The results show that high level of miR-937-3p can reduce the influence of si-MYC on the invasion and metastasis of LUAD cells (Fig. 6I, J, Additional file 3: Fig. S3B, C).

## Discussion

With the improvement of the detection level, the incidence of lung cancer is getting higher and higher. Studies have shown that miRNAs can be used as biomarkers for malignant tumors to determine prognosis and guide treatment [12]. Most deaths from lung cancer are due to metastasis and metastasis-related complications [13]. When tumor expansion occurs, internal tumor cells will move away from their blood supply and become relatively hypoxic. Hypoxia can promote the expression of multiple angiogenic factors in tumor cells [14–17]. Angiogenesis forms the pre-metastasis microenvironment, thereby promoting the distant metastasis of the tumor.

We first found that miR-937-3p is highly expressed in lung adenocarcinoma in the TCGA database. Studies have found that miR-937-3p is up-regulated in breast cancer, and high miR-937-3p expression is associated with a weaker survival rate in cancer patients [18]. Recent studies have shown that miR-937-3p has a guiding role in the prognosis and diagnosis of breast cancer. Inhibition of miR-937-3p expression may be a new type of targeted therapy for breast cancer [19].We have further verified on 60 specimens of LUAD patients, and miR-937-3p is significantly higher in cancer tissues than in adjacent tissues. The most important thing is that we found that the expression of miR-937-3p is related to tumor metastasis. miR-937-3p was significantly higher than 16HBE in LUAD cell lines, and ISH showed that miR-937-3p was mainly expressed in malignant tumors. Vitro experiments have showed that the overexpression of miR-937-3p can promote angiogenesis, invasion and metastasis of LUAD cells, while the inhibition of miR-937-3p has the opposite effect. Western blot results showed that the up-regulation of miR-937-3p significantly increased the expression of N-cadherin, vimentin and slug, and decreased the expression of E-cadherin. In vivo, we found that miR-937-3p promotes the metastasis of xenograft tumors. Therefore, we speculate that miR-937-3p has played a role in promoting tumor progression in the development of LUAD.

**Fig. 7:**
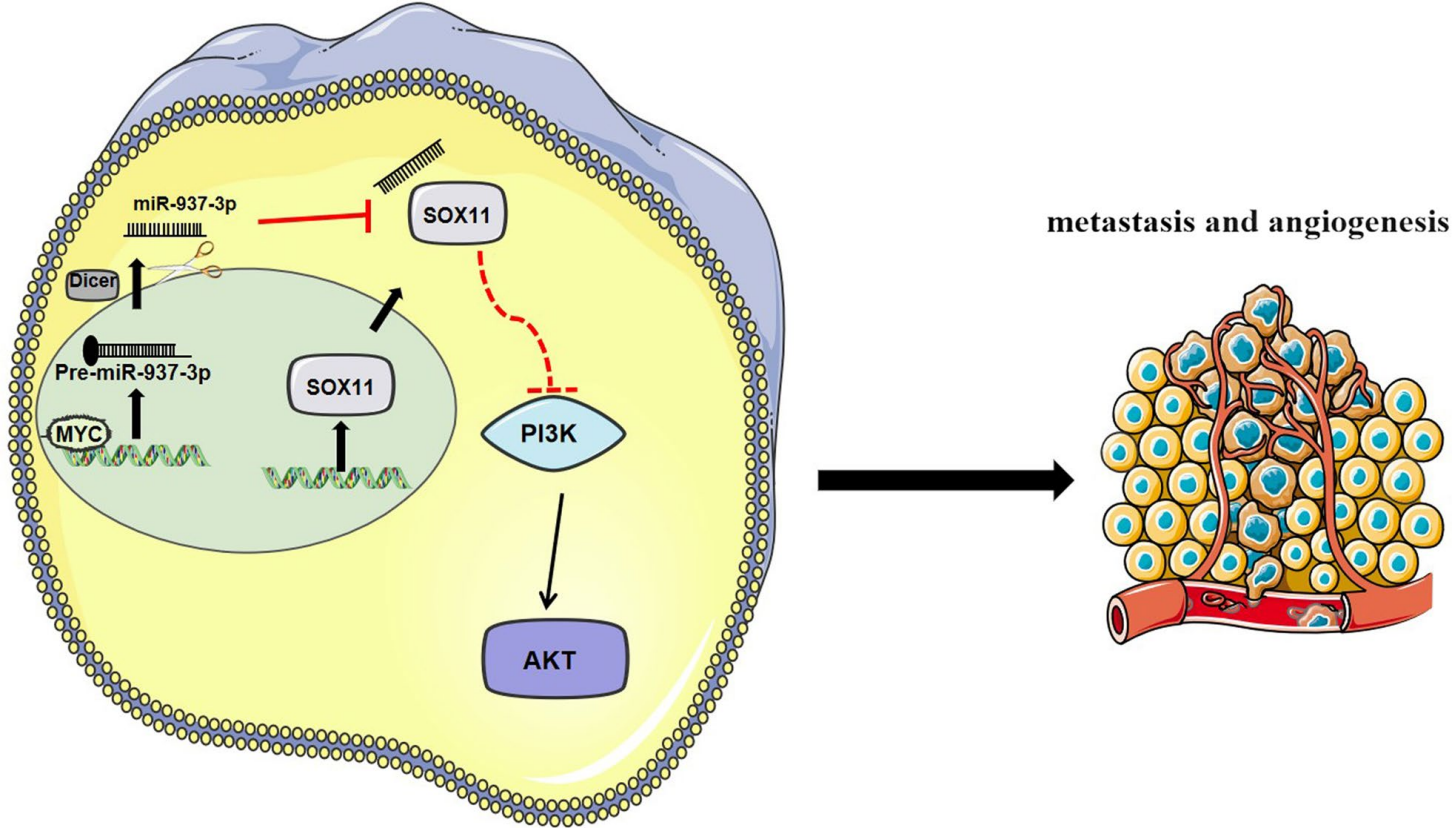
Schematic diagram of MYC/miR-937-3p/SOX11/PI3K/AKT pathway

The miRNA can bind to the target gene 3′-UTR and inhibit the expression of the target gene. For example, miR-665 can promote breast cancer metastasis by acting on NR4A3 [20]. We predicted through 3 websites (miRDB, Targetscan and miRWalk) that SOX11 is a target gene of miR-937-3p. SOX11 belongs to the C group of mammalian SOX proteins [21]. SOX11 was first reported in lymphoma [22]. SOX11 is a diagnostic and prognostic antigen of B-cell lymphoma [23, 24], and has been shown to have tumor suppressor function. However, there are very few studies on the expression and significance of SOX11 in lung cancer. SOX11 promotes apoptosis of liver cancer cells through Wnt signaling pathway [25]. Overexpression of SOX11 can inhibit the invasion of colon cancer [26]. Through luciferase reporter gene experiments and Western blotting analysis, we found that SOX11 is the target gene of miR-937-3p. In addition, we found through western blot that miR-937-3p can target SOX11 to activate the PI3K/AKT pathway, thereby promoting NSCLC angiogenesis and mediating NSCLC metastasis. Therefore, we speculate that miR-937-3p can promote the angiogenesis and metastasis of LUAD by targeting SOX11, which has been verified by in vivo and in animal experiments (Fig. 7).

In addition, miRNAs can be regulated by upstream transcription factors and are highly expressed in malignant tumors. Using JASPAR and UCSC databases, we found that MYC is an upstream regulator of miR-937-3p. MYC is a transcription factor and is considered to be one of the most frequently out of control oncogenes [27]. It is frequently translocated in cancers of the hematopoietic system, and was found to be the third most amplified gene in human cancers in whole cancer copy number analysis [28, 29]. In gastric cancer, OCT4 and MYC can bind to the promoter region of miR-9 to trigger its transcription [30]. Several recurring genetic aberrations including MYC have been found in small cell lung cancer (SCLC). Therefore, MYC family genes are the driving factors of carcinogenesis and may soon become new therapeutic targets [31–33]. But the role of MYC in LUAD needs further study. In our study, ChIP analysis showed that MYC binds to a binding site upstream of miR-937-3p. Down-regulation of MYC has resulted in reduced miR-937-3p expression and increased SOX11 expression, suggesting that there is an axis between MYC/miR-937-3p/SOX11 signaling. It was further verified by rescue experiments, showing that MYC can promote angiogenesis, invasion and metastasis by regulating miR-937-3p transcription.

## Conclusions

Our research shows that miR-937-3p is overexpressed in LUAD tissues and is associated with metastasis. miR-937-3p is regulated by MYC, and miR-937-3p can promote the angiogenesis, invasion and metastasis of LUAD by binding to the 3′-UTR of SOX11. We have provided a new perspective on the regulation of LUAD, and which may be a potential treatment for LUAD.

## Supplementary Information

**Additional file 1: Fig. S1.** Differential expression of miRNAs in lung adenocarcinoma and adjacent tissues.

**Additional file 2: Fig. S2.** (A) The miRNA (miR-195 miR-142 and miR-345, *Fig. 1B*) were closely related with survival probability. (B) miRNA (miR-937-3p, miR-195 miR-142 and miR-345,) expression in metastatic and non-metastatic tissues. (C) After treatment and the expression level of miR-937-3p in HUVECs was detected by qRT-PCR. (D) After transfection and the expression level of MYC was detected by qRT-PCR. (E) According to JASPAR database, the prediction score of MYC binding to miR-937-3p was the highest.

**Additional file 3: Fig. S3.** (A) Levels of miR-937-3p and SOX11 was accessed by qRT-PCR after co-transfection. (B)(C)migration and invasion was determined after co-transfected; scale bars, 20 μm; scale bars, 200 μm. The data expressed as the mean ± SD. (*P < 0.05; **P < 0.01; ***P < 0.001).

**Additional file 4: Table S1.** Sequences of the primers in this study.

## Data Availability

The datasets used and/or analyzed during the current study are available from the corresponding author on reasonable request.
