# Supplementary figures and images for: MiR-937-3p promotes metastasis and angiogenesis and is activated by MYC in lung adenocarcinoma
Source: Cancer Cell Int. 2022 Jan 15;22:31. doi: 10.1186/s12935-022-02453-w (PMC8761314; doi:10.1186/s12935-022-02453-w)

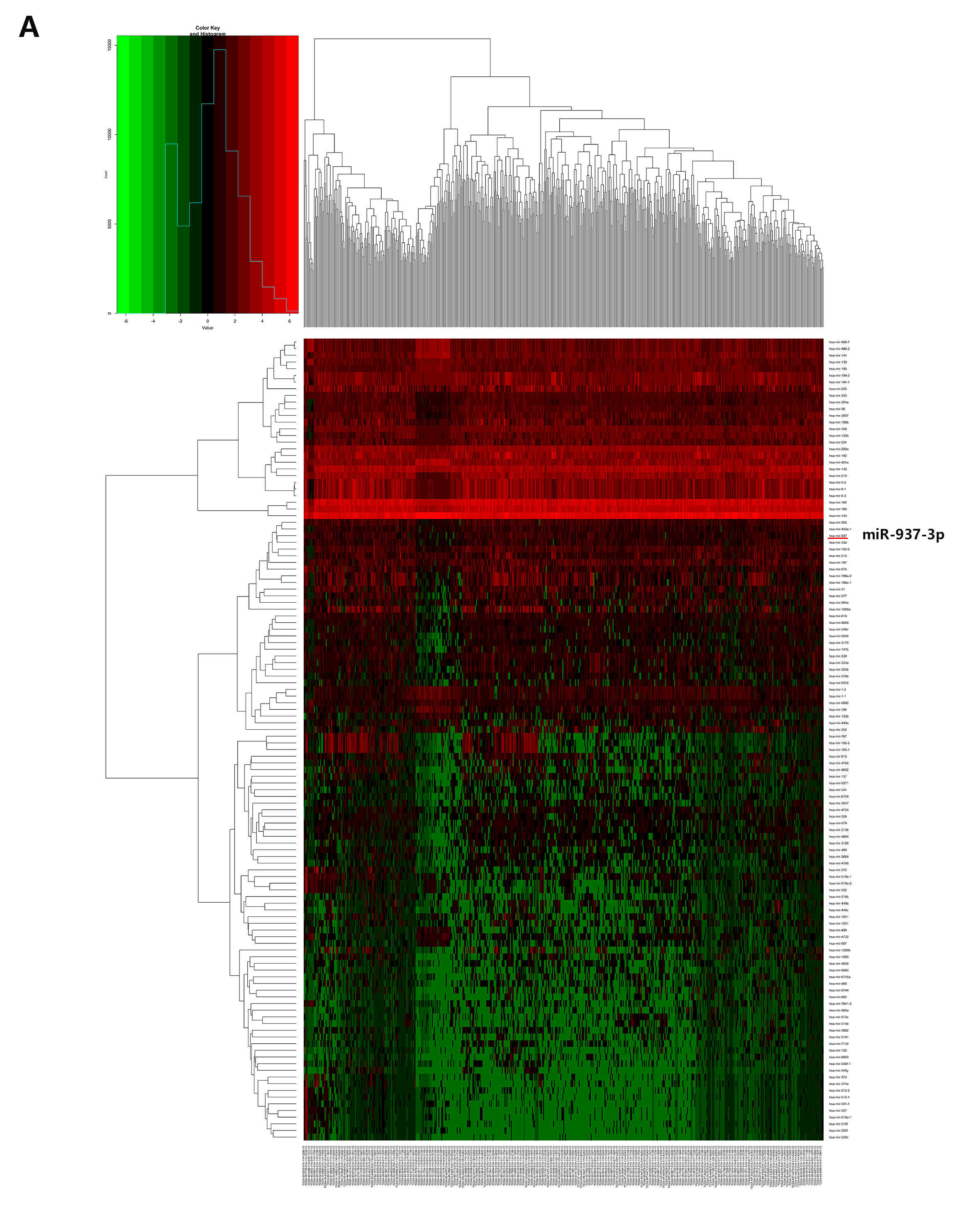

Supplement: Supplementary file 1 — Additional file 1: Fig. S1. Differential expression of miRNAs in lung adenocarcinoma and adjacent tissues. [file 12935_2022_2453_MOESM1_ESM.tif]

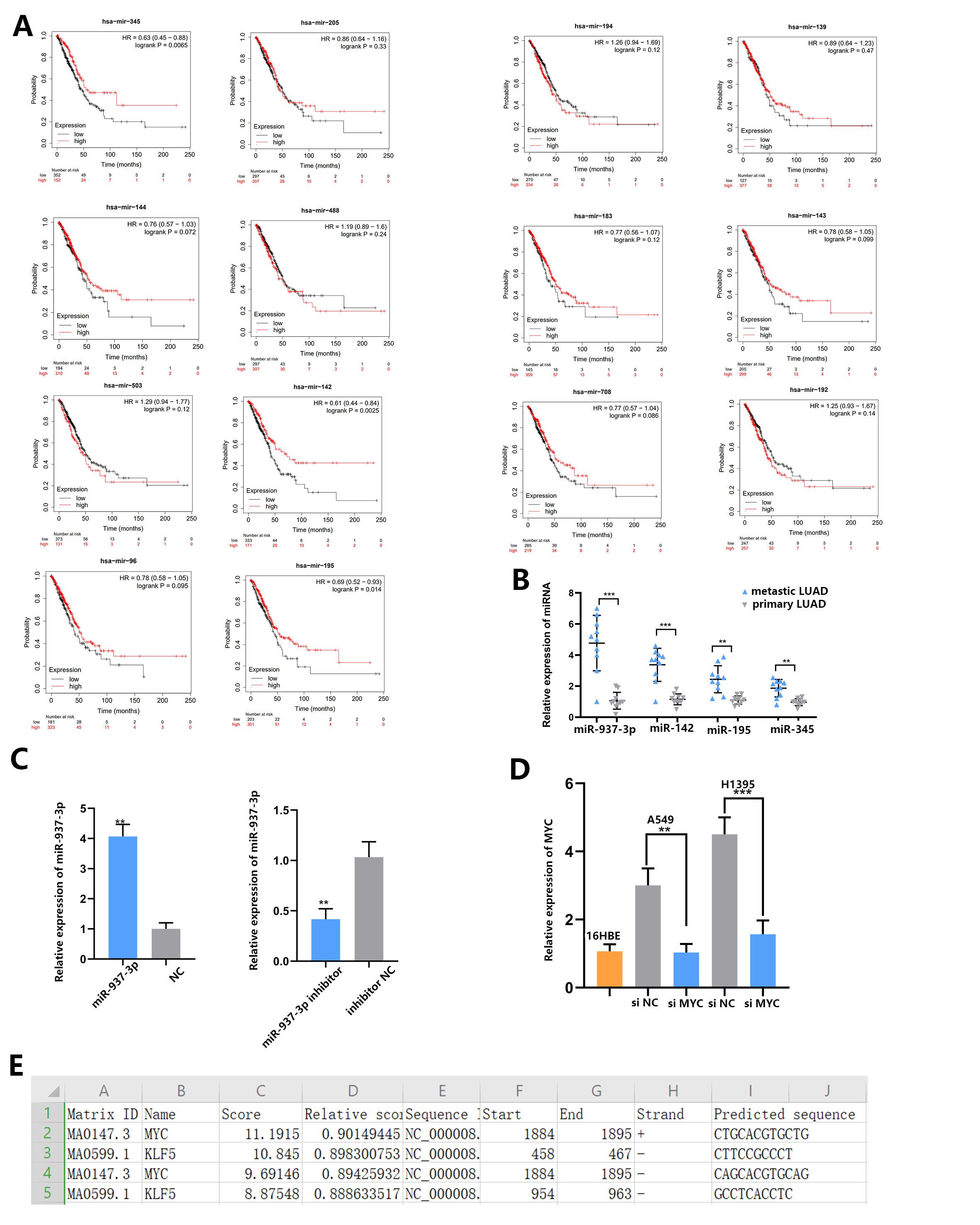

Supplement: Supplementary file 2 — Additional file 2: Fig. S2. (A) The miRNA (miR-195 miR-142 and miR-345, Fig. 1B) were closely related with survival probability. (B) miRNA (miR-937-3p, miR-195 miR-142 and miR-345,) expression in metastatic and non-metastatic tissues. (C) After treatment and the expression level of miR-937-3p in HUVECs was detected by qRT-PCR. (D) After transfection and the expression level of MYC was detected by qRT-PCR. (E) According to JASPAR database, the prediction score of MYC binding to miR-937-3p was the highest. [file 12935_2022_2453_MOESM2_ESM.tif]

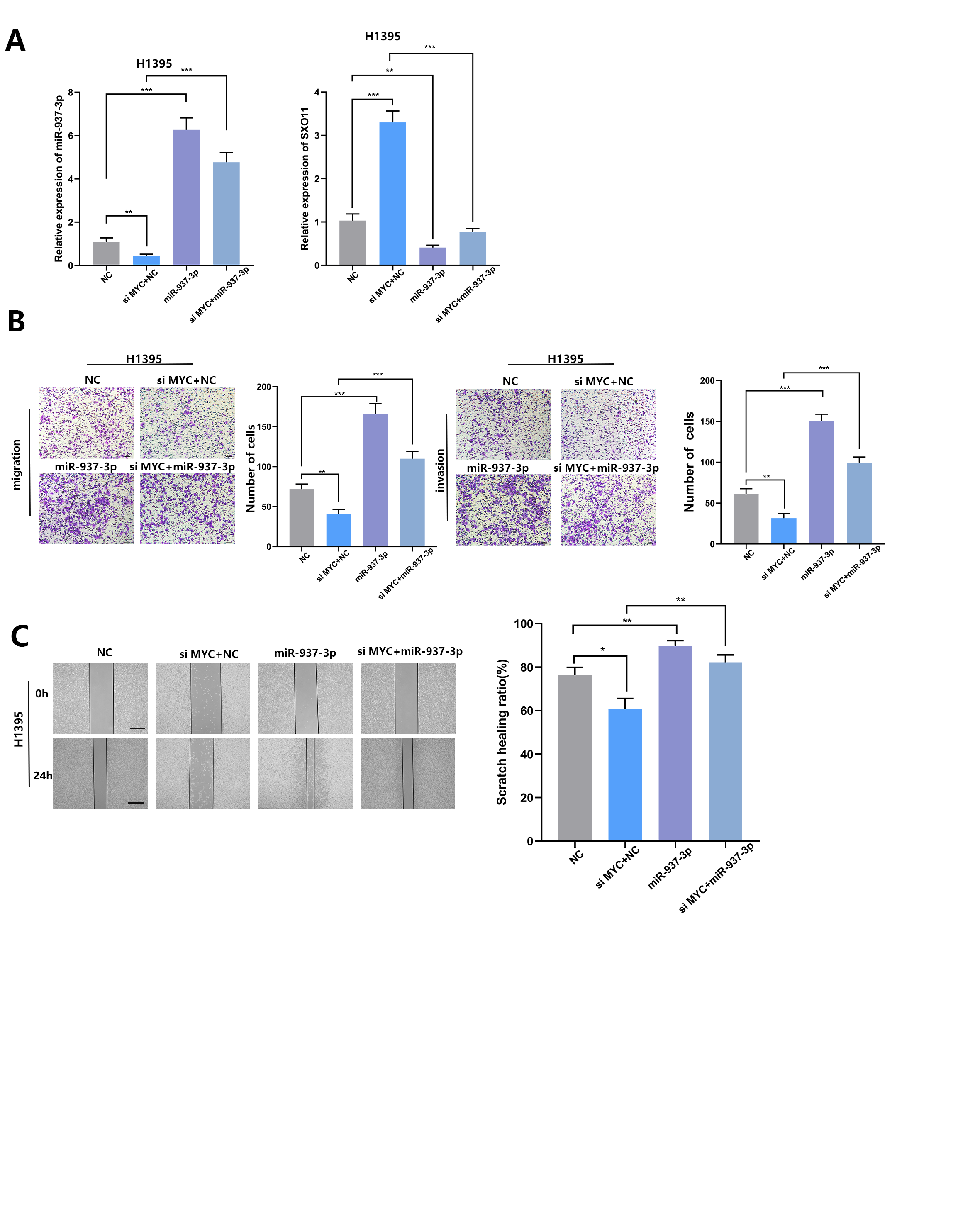

Supplement: Supplementary file 3 — Additional file 3: Fig. S3. (A) Levels of miR-937-3p and SOX11 was accessed by qRT-PCR after co-transfection. (B)(C)migration and invasion was determined after co-transfected; scale bars, 20 μm; scale bars, 200 μm. The data expressed as the mean ± SD. (*P < 0.05; **P < 0.01; ***P < 0.001). [file 12935_2022_2453_MOESM3_ESM.tif]
