# Supplementary material for: MiR-937-3p promotes metastasis and angiogenesis and is activated by MYC in lung adenocarcinoma
Source: Cancer Cell Int. 2022 Jan 15;22:31. doi: 10.1186/s12935-022-02453-w (PMC8761314; doi:10.1186/s12935-022-02453-w)
Supplement: Supplementary file 4 — Additional file 4: Table S1. Sequences of the primers in this study. [file 12935_2022_2453_MOESM4_ESM.docx]

Supplementary table 1 | Sequences of the primers in this study

| Name | Sequence (5′ → 3′) |
| --- | --- |
| miR-937-3p mimics | AUCCGCGCUCUGACUCUCUGCC |
| miR-937-3p mimics NC | ACUAUCGAGUGACCGUAGAA |
| miR-937-3p inhibitor | GGCAGAGAGUCAGAGCGCGGAU |
| miR-937-3p inhibitor NC | AUCUCCGAACGUGUCACGUA |
| ISH of miR-937-3p | AUCCGCGCUCUGACUCUCUGCC |
